# Effect of Protection Polymer Coatings on the Performance of an Amperometric Galactose Biosensor in Human Plasma

**DOI:** 10.3390/bios14040167

**Published:** 2024-03-30

**Authors:** Carina Figueiredo, Carolin Psotta, Kavita Jayakumar, Anna Lielpetere, Tanushree Mandal, Wolfgang Schuhmann, Dónal Leech, Magnus Falk, Marcos Pita, Sergey Shleev, Antonio L. De Lacey

**Affiliations:** 1 Instituto de Catálisis y Petroleoquímica, CSIC, c/Marie Curie 2, 28049 Madrid, Spain; carina.felix@estudiante.uam.es (C.F.);; 2 Department of Biomedical Science, Faculty of Health and Society, & Biofilms-Research Center for Biointerfaces, Malmo University, 205 06 Malmö, Swedensergey.shleev@mau.se (S.S.); 3 Aptusens AB, 293 94 Kyrkhult, Sweden; 4 School of Biological and Chemical Sciences & Ryan Institute, University of Galway, H91 TK33 Galway, Irelandtanushreemandal2014@gmail.com (T.M.);; 5 Analytical Chemistry-Center for Electrochemical Science (CES), Faculty of Chemistry and Biochemistry, Ruhr-University Bochum, 44791 Bochum, Germany; anna.lielpetere@ruhr-uni-bochum.de (A.L.); wolfgang.schuhmann@rub.de (W.S.)

**Keywords:** galactose, biosensor, plasma, protection polymers

## Abstract

Galactose monitoring in individuals allows the prevention of harsh health conditions related to hereditary metabolic diseases like galactosemia. Current methods of galactose detection need development to obtain cheaper, more reliable, and more specific sensors. Enzyme-containing amperometric sensors based on galactose oxidase activity are a promising approach, which can be enhanced by means of their inclusion in a redox polymer coating. This strategy simultaneously allows the immobilization of the biocatalyst to the electroactive surface and hosts the electron shuttling units. An additional deposition of capping polymers prevents external interferences like ascorbic or uric acid as well as biofouling when measuring in physiological fuels. This work studies the protection effect of poly(2-methacryloyloxyethyl phosphorylcholine-*co*-glycidyl methacrylate (MPC) and polyvinylimidazole-polysulfostyrene (P(VI-SS)) when incorporated in the biosensor design for the detection of galactose in human plasma.

## 1. Introduction

One of the greatest challenges in healthcare is the real-time diagnosis of diseases by direct and fast analysis in physiological fluids. Biosensors emerge as a solution for this goal [1]. In particular, amperometric enzyme biosensors have several advantages for monitoring analytes of clinical importance. The electrochemical transducers allow high sensitivity, fast time response, design simplicity, and low cost of the device, while the immobilized enzyme provides the required selectivity for the detection of the target analyte [1,2,3,4]. The microfabrication of sensors and the use of hybrid biomaterials help to improve the biocompatibility of these devices in long-term use [5,6,7]. Global efforts to ensure quality implantable devices are expanding, leading to a growth rate in the biosensors market [8]. Close attention to all possible medical device trends is further highlighted by the proliferation of personalized medicine, point-of-care testing, as well as wearable devices [9]. However, physiological fluids are complex solutions that pose several challenges for direct measurement with amperometric enzyme biosensors. They contain several electrochemically active interferences as well as biological entities (cells, proteins, etc.) that cause passivation of the electrode or inactivation of the immobilized enzyme [10]. Despite the evident medical benefits related to the continuous in-vivo measurement of glucose levels in blood by commercial biosensors, there are major challenges to make the implanted biosensors work in the long term [6,8,9]. The presence of ascorbic and uric acids in blood causes measurement interferences due to their oxidation at the electrode when poised at relatively moderate potentials [11]. In addition, signal degradation and biosensor reliability over time are common and attributed to biofouling (non-specific cell and protein absorption) [12,13]. Device implantation triggers a cascade of inflammatory responses in the body, culminating in fibrosis and collagen encapsulation on the implanted materials. This process, known as foreign body response (FBR), largely affects the biosensor function, leading to gradual loss of the biosensor sensitivity and, finally, to complete loss of function [14,15].

Cutting edge research in biomimetic materials has been explored to overcome these drawbacks. Negatively charged polymers covering the biocatalytic layer on the electrode have been shown to be useful for minimizing the interference signal from ascorbic and uric acids [16,17]. Antifouling strategies to achieve more biocompatible biosensors have been implemented by incorporating hydrophilic polymers as a protection layer. Polyethylene glycol (PEG) has been widely studied for this purpose [18,19]. Combinations of polymers have also been studied in order to obtain less adhesion of proteins and cells on the surface while causing minimal interference on the implanted device output [20,21]. Synergistic behavior between device operability and reduced inflammatory response has been reported by using zwitterionic polymer coatings. They possess stronger hydration effects that result in enhanced anti-fouling properties [22,23,24]. Very recently, Jayakumar et al. reported a comparison between several coatings for minimizing FBR over an amperometric glucose biosensor. The best results were obtained with poly(2-methacryloyloxyethyl phosphorylcholine-*co*-glycidyl) methacrylate (MPC). MPC reduced the adhesion of fibroblast and fibrinogen by 80% and 50%, respectively, without loss of glucose detection sensitivity [25]. Although it was only tested for a glucose biosensor, this study opens the door for the development of other biosensors to be used in physiological fluids in order to decrease the impact of FBR over them.

Quantitative determination of galactose in blood or urine is also clinically important. Increased levels of this sugar are caused by galactosemia, which is a disease characterized by the inability to metabolize the monosaccharide galactose [26,27]. Galoctosemia is especially important in newborn infants and young children, since galactose is produced by the hydrolysis of the lactose present in most animal milks [28]. Increased concentrations of galactose and galactose-1-phosphate caused by this illness provoke bleeding disorders, sepsis, cataracts, and even death [29,30,31]. Like other diseases, the timetable for discovery is essential to avoid major complications. Therefore, the development of biosensors to avoid the use of expensive and time-consuming quantification methods is desirable. In the literature, there are several reports of electrodes with immobilized galactose oxidase (GaOx) tested as amperometric galactose biosensors in serum or plasma [32,33]. The transduction signal of these biosensors is either based on the change in the current due to O_2_ uptake [16] or H_2_O_2_ production [34] by the enzymatic activity or by adding a redox mediator as electron acceptor from GaOx instead of O_2_ [35,36]. We recently reported an amperometric biosensor for determination of galactose in dairy products not affected by the presence of lactose or glucose based on galactose oxidase (GaOx) co-immobilized with an osmium complex modified redox polymer on glassy carbon electrodes. This biosensor configuration has two main advantages. The first one is that GaOx is very efficiently wired by the Os complex modified redox polymer, thus outcompeting O_2_ as an electron acceptor and yielding an O_2_-insensitive galactose biosensor. The second advantage is that the activity of the immobilized GaOx can be controlled by the redox potential applied at the electrode, which switches it on or off by oxidizing GaOx to the active state or reducing it to the inactive state [37].

We now study the performance of the electrodes modified with GaOx and the Os complex modified redox polymer for detection of galactose in human plasma. To decrease the interferences and inactivation effects of the physiological medium, we added a coating system to our modified electrode. The coating system was previously developed for a glucose biosensor tested in a buffer solution mimicking plasma [38] and consists of: (i) an interlayer of a negatively charged polyvinylimidazole-polysulfostyrene co-polymer, denoted as P(VI-SS), for protection against uric and ascorbic acids; and (ii) an outer zwitterionic polymer coating of MPC for preventing cell and protein adhesion. Results of galactose detection in plasma with this biosensor configuration were compared with those obtained with uncoated GaOx-modified electrodes.

## 2. Materials and Methods

### 2.1. Chemicals

All chemicals were of analytical grade and used as received. D-(+)-galactose, sodium dihydrogen, and poly(ethylene glycol)diglycidyl ether (PEGDGE, MW 500) were supplied by Sigma-Aldrich (St. Louis, MI, USA). Di-sodium hydrogen phosphate 12-hydrate was obtained from VWR Chemicals (Solon, OH, USA). Micropolish alumina with 1, 0.3, and 0.05 µm particle sizes were purchased from Buehler. The redox polymer [poly(1-vinylimidazole) Os(2,2′-bipyridine)_2_Cl]^+^, named as PVI-Os, was synthesized by modification of reported methods [39,40]. P(VI-SS) was synthesized as reported by Lielpetere et al. [38]. MPC was synthesized as described by Jayakumar et al. [25]. Galactose oxidase (GaOx) from *Dactlylium dendroides* (500–1500 U mg^−1^) was supplied by Sigma-Aldrich. Human plasma was received from Skånes Universitetssjukhus (Lund, Sweden). All aqueous solutions were prepared with ultrapure deionized water (18.2 MΩ cm) from a Milli-Q water system.

### 2.2. Electrode Modification

Glassy carbon electrodes from BASi (3 mm diameter) were polished using sandpaper (P1500) and alumina of decreasing sizes (1, 0.3, 0.05 µm). Afterward, they were sonicated in Milli-Q grade water for 15 min, rinsed, and dried. Two types of electrode modification were done on the clean surfaces, named as uncoated and coated. In the first case, the electrode surface was modified by drop-casting with 14 µL of an aqueous solution containing 5 mg mL^−1^ PVI-Os, 0.10 mg mL^−1^ PEGDGE, and 1 mg mL^−1^ GaOx. The deposited drop was allowed to dry overnight. In the case of the coated electrodes, after doing the first modification step as the uncoated electrodes, the electrodes were modified with two coating polymers. First, 5 µL of 0.5 wt/v% P(VI-SS) were deposited, allowing 2 h for this deposit to dry, and after this, 5 µL of 0.5 wt/v% MPC were deposited with overnight drying at ambient temperature.

### 2.3. Electrochemical Measurements

All experiments were performed in a three-electrode configuration using a glassy carbon-modified electrode as the working electrode, a Pt wire as a counter electrode, and a custom-built Ag|AgCl (3 M KCl) as a reference electrode. All redox potentials mentioned are relative to this reference electrode. The electrochemical experiments were performed using a μAutolab Type III/FRA2 potentiostat/galvanostat from Metrohm Autolab B.V. (Utrech, The Netherlands). Measurements were performed using either a 0.1 M phosphate buffer, pH 7.0, or human plasma. The plasma was received from Skånes Universitetssjukhus (Lund, Sweden). For characterization in the physiological medium, the measurement solution was heated to 37 °C and continuously stirred. During chronoamperometric measurements, the applied potential was +350 mV, and magnetic stirring of the electrolyte was performed to ensure a homogeneous solution after the addition of different substrate concentrations. All chronoamperometric measurements were performed under ambient air conditions. For the cyclic voltammetry measurements, the potential was scanned from 0 to +450 mV at 5 or 10 mV s^−1^ scan rates. Current signals were normalized to the geometric surface area (0.07 cm^2^) of the glassy carbon electrodes to generate current density data.

For the modified electrodes tests with plasma, a sequence of experiments was performed to mimic a flow cell system. The repetition of steps was as follows: (a) electrode immersion in 0.1 M phosphate buffer, pH 7; (b) spiking the electrolyte with 45 mM galactose; (c) electrode immersion in 0.1 M phosphate buffer, pH 7; (d) electrode immersion in human plasma; (e) spiking the plasma with 45 mM galactose; (f) electrode in human plasma; (g) electrode immersion in 0.1 M phosphate buffer, pH 7; (h) spiking with 45 mM galactose; (i) electrode immersion in 0.1 M phosphate buffer, pH 7. The duration of each step was about 500 s, and magnetic stirring was done to ensure a homogeneous solution after the addition of different substrate concentrations.

## 3. Results and Discussion

**Figure 1:**
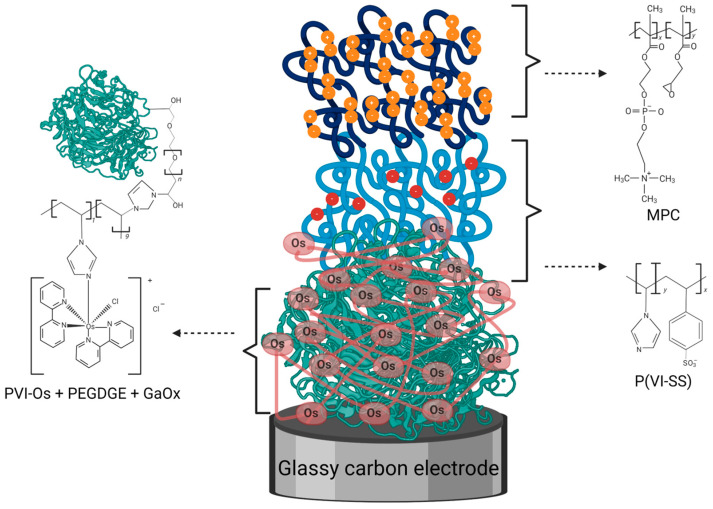
Schematic illustration of a coated galactose biosensor comprising a glassy carbon electrode modified with a bottom layer of GaOx, PEGDGE crosslinker, and PVI-Os, a middle layer of P(VI-SS) at a 1:1 monomer ratio, and a top layer of MPC with a 30% loading of glycidyl methacrylate monomer.

We prepared amperometric galactose biosensors by co-immobilization of galactose oxidase (GaOx) from *Dactylium dendroides* and an osmium-based redox polymer (PVI-Os) on a glassy carbon electrode using PEGDGE as crosslinker [37] and studying their performance in human plasma. As illustrated in Figure 1, in some of the modified electrodes, two additional polymers were added on top as a protection layer: the negatively charged polymer P(VI-SS) for protection against the electrochemical interferents uric and ascorbic acid and an outer zwitterionic polymer coating of MPC for antifouling protection using layers that can be cross-linked to each other to minimize the boundary between them. This coating configuration was previously shown to be optimal for protecting a glucose biosensor based on cellobiose dehydrogenase operating in plasma-mimicking buffer [38], and we now study it in real human plasma for a galactose biosensor based on the enzyme GaOx. The structure and catalytic mechanism of GaOx are very different from those of cellobiose dehydrogenase [33,41]. Thus, its inhibition conditions as well as operational and storage stabilities in physiological media are expected to be quite different.

**Figure 2:**
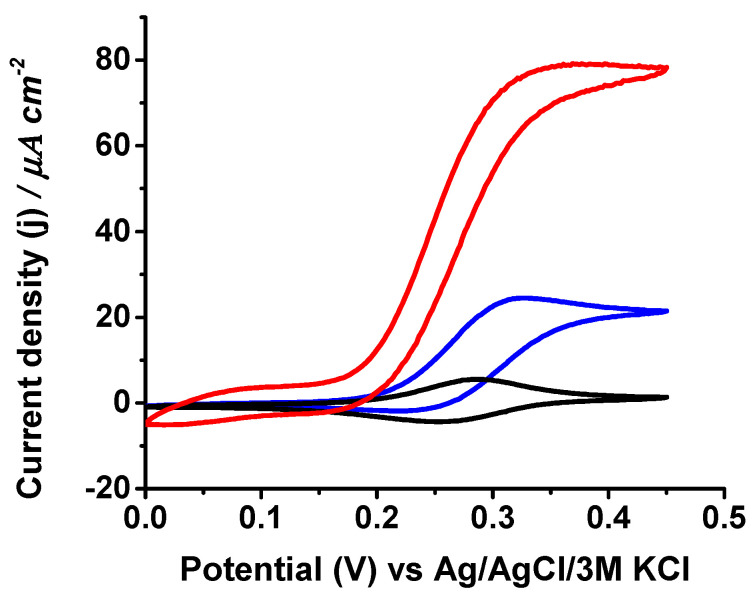
Cyclic voltammograms of uncoated (red) and coated (blue) GaOx-modified electrodes measured at 10 mV s^−1^ scan rate in 0.1 M phosphate buffer pH 7.0 containing 100 mM galactose. The black line corresponds to the CV in absence of galactose for the uncoated electrode.

First, the electrochemical response of uncoated and coated GaOx-modified electrodes to galactose in pH 7.0 phosphate buffer was studied. Although the electrocatalytic currents of galactose oxidase increase considerably in basic solutions [42], for comparison with measurements done in physiological samples, we chose neutral pH as the standard value for evaluating the biosensor performance. Figure 2 shows the expected cyclic voltammograms showing the quasi-reversible redox process of the immobilized PVI-Os polymer in the absence of the substrate and the electrocatalytic effect upon galactose addition to the electrolyte measured for both uncoated and coated electrodes.

**Figure 3:**
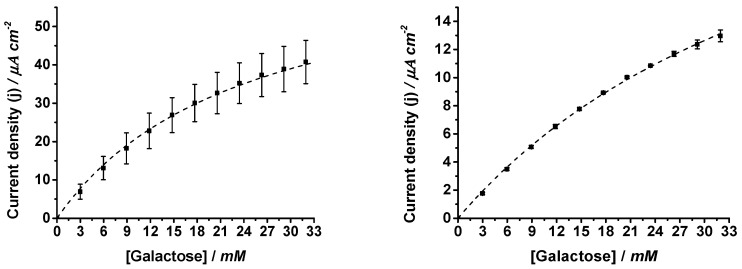
Current density dependence on galactose concentrations for uncoated (**left graph**) and coated GaOx-modified electrodes (**right graph**) measured by chronoamperometry measurements at 0.35 V vs. Ag/AgCl (3 M KCl) in 0.1 M phosphate buffer pH 7.0. Error bars refer to the standard deviation (n = 3).

Chronoamperometric experiments at +350 mV vs. Ag/AgCl (3 M KCl) were also performed, in which increasing galactose concentrations were added to the 0.1 M phosphate buffer with pH 7 electrolyte (Figure 3). This applied potential was selected because it provides a sufficient overpotential to assure the oxidation by the co-immobilized Os complex of the tyrosine of the enzyme’s active site, which keeps it in the active state. A lower redox potential would decrease the galactose oxidase activity due to formation of the inactive state, whereas a higher applied potential would increase the oxidative interference currents [37]. The catalytic currents measured with the coated electrodes were approximately one third of those measured with the uncoated ones at the same galactose concentration, although the linear range of response was broader. Both effects can be explained by the additional diffusional constraint of substrate access to the immobilized enzyme due to the protection layers, P(VI-SS) and MPC [43,44].

**Figure 4:**
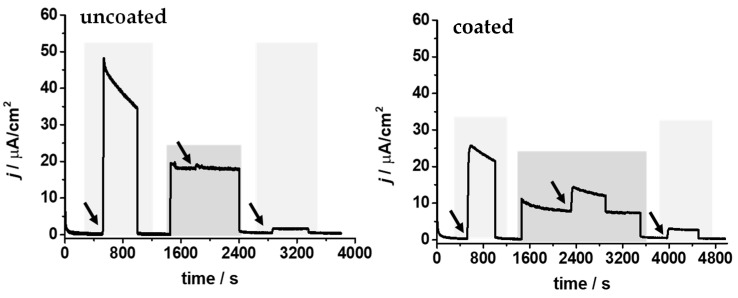
Chronoamperometry curves performed at 0.35 V vs. Ag/AgCl (3 M KCl) with an uncoated (**left graph**) and a coated (**right graph**) biosensor. The electrolyte was changed sequentially from 100 mM phosphate buffer pH 7.0 (light grey) to human plasma (dark grey). The addition of 45 mM galactose is marked by arrows.

Both types of GaOx-modified electrodes were tested for galactose detection in the physiological medium (Figure 4). Firstly, 100 mM of phosphate buffer pH 7.0 was used as the electrolyte, and, after background current stabilization, 45 mM of galactose was added as the reference value. The measured currents reached 48 µA cm^−2^ and 26 µA cm^−2^ for the uncoated and coated electrodes, respectively. In a second step, the electrolyte was exchanged for human plasma. The background current increased considerably in the case of the uncoated electrode due to the oxidation of the interferences ascorbic and uric acid at the electrode, reaching 20 µA cm^−2^. When 45 mM of galactose was added, the increase in the current was negligible compared to the background currents. Moreover, after reintroducing the phosphate buffer, the background current returned to its low initial value of 1 µA cm^−2^, but the catalytic current in the presence of 45 mM of galactose was only approximately 5% of the initial one. This result clearly indicated that the plasma medium was too harsh for GaOx, irreversibly losing most of its enzymatic activity. On the other hand, the coated biosensor yielded a background current in human plasma that was half of that measured with the uncoated electrode, confirming the effect of the P(VI-SS) polymer in decreasing the interference of ascorbic and uric acids, as studied for a glucose biosensor by Lielpetere et al. [38]. This effect is attributed to the high loading of sulfonate groups in P(VI-SS), causing electrostatic repulsion of negatively charged interferents. Thus, it prevents the interference of ascorbic and uric acids while not affecting the difussion of monosacharides, such as glucose or galactose, contrary to other negatively charged polymers like Nafion [38]. Furthermore, approximately 33% of the electrocatalytic response with 45 mM of galactose was maintained in human plasma compared to the one measured in phosphate buffer. Therefore, the combined P(VI-SS) and MPC capping polymers layer was preventing complete passivation of the biosensor in the physiological fluid, thus allowing galactose detection in human plasma. However, the operational stability was low, as, after 1 h, the response in buffer decreased by 90%.

**Figure 5:**
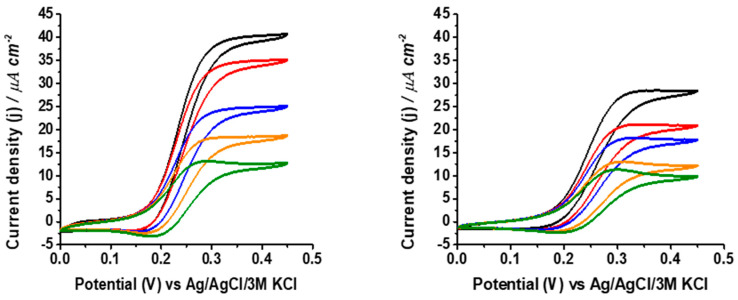
Cyclic voltammetry measurements of uncoated (**left graph**) and coated (**right graph**) GaOx-modified electrodes measured at 5 mV s^−1^ scan rate in 0.1 M phosphate buffer pH 7.0 containing 45 mM galactose after 1 (black), 2 (red), 3 (blue), 4 (orange), and 7 days (green) stored overnight in human plasma at 4 °C.

**Figure 6:**
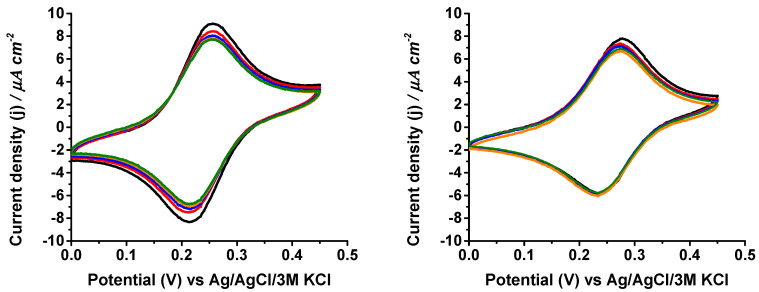
Cyclic voltammetry measurements of uncoated (**left graph**) and coated (**right graph**) GaOx-modified electrodes measured at 5 mV s^−1^ scan rate in 0.1 M phosphate buffer pH 7.0 after stored overnight in human plasma at 4 °C for 1 to 7 days. The colors code is as in Figure 5.

The storage stability of both coated and uncoated electrodes was also studied by cyclic voltammetry and chronoamperometry. The biosensors’ responses to increasing galactose concentrations were repeatedly measured in 100 mM phosphate buffer during several days, which was kept in the fridge overnight in plasma to test the operability of the biodevices under adverse storage conditions. Both systems gradually lost electrocatalytic response to galactose each day, although the electrode passivation by the plasma was slightly higher in the uncoated electrodes (Figure 5). The coated biosensor still had 28% of the initial response after one week. The chronoamperometry measurements also showed the same trend during an equal period of time (Figure S1, Supplementary Materials). The loss of electrocatalytic activity of galactose oxidation was not caused by leakage of the PVI-Os from the electrode surface, because, by cyclic voltammetry in buffer and in the absence of the substrate, it was checked that the peak currents of the redox polymer after 7 days were 78% and 77% for the coated and uncoated electrodes, respectively (Figure 6).

The cyclic voltammetry and chronoamperometry measurements show that, when the galactose biosensor was stored in human plasma, the loss of GaOx activity was much slower (retaining significant electroactivity after 7 days, even without the protection polymers), whereas, under operation conditions, the loss of activity of the uncoated biosensor was drastic within a few minutes. Therefore, these results suggest that the GaOx is much more vulnerable to irreversible inactivation in the physiological medium when in its active oxidized state, as, under operational conditions of galactose oxidation, the enzyme is poised at a redox potential in which both its Cu atom and the tyrosine residue of the catalytic site are oxidized [33,37,45]. The presence of the two capping polymers is then essential as a protection layer to prevent complete and rapid inactivation of the GaOx catalytic site. When stored in human plasma, the GaOx was in the intermediate inactive state with its catalytic Tyr reduced and the oxidized Cu^2+^, as the final potential of the cyclic voltammetry measurements was 0 V vs. Ag/AgCl (3M KCl) [33,45]. In this case, the loss of activity was much slower, even without the protection polymers. Thus, our results suggest that inactivation of GaOx in plasma is caused by the interaction of one of the plasma components with the tyrosine radical of the enzyme’s active site, and that coating of the galactose biosensor is required to prevent against such inactivation.

## 4. Conclusions

The deposition of polymers MPC and P(VI-SS) as capping layers over the biosensor based on GaOx crosslinked with the PVI-Os redox polymer allows detection of galactose in human plasma. The protection effects of the capping polymers are twofold. They greatly decrease the interference of ascorbic and uric acids present in the physiological medium, and they mitigate, but not preclude completely, the total, fast, and irreversible inactivation of GaOx observed for the uncoated electrode when measuring galactose in human plasma. The comparison between operational and storage stability experiments in human plasma for both uncoated and coated biosensors indicates that the immobilized GaOx is much more vulnerable to irreversible inactivation when in the active oxidized state than in the inactive intermediate state. As the redox state of the active center of the immobilized GaOx can be controlled by its wiring to the electrode with the co-immobilized PVI-Os redox polymer, it would be possible to switch on the coated biosensor for fast measurements of galactose in plasma and then switch it off in the periods between measurements to increase its stability.

The results of this work suggest that the inactivation of GaOx in plasma is caused by the interaction of one of its components with the tyrosine radical of the enzyme when it is in the oxidized active state, although confirmation of this hypothesis and identification of this component causing irreversible inactivation requires future work to obtain direct experimental evidence by other techniques.

## Data Availability

The data presented in this study are available on request from the corresponding author.

## Supplementary Materials

The following supporting information can be downloaded at: https://www.mdpi.com/article/10.3390/bios14040167/s1, Figure S1: Storage stability of uncoated and coated biosensors studied by chronoamperometry.
