# Supplementary material for: Effect of Protection Polymer Coatings on the Performance of an Amperometric Galactose Biosensor in Human Plasma
Source: Biosensors (Basel). 2024 Mar 30;14(4):167. doi: 10.3390/bios14040167 (PMC11047878; doi:10.3390/bios14040167)
Supplement: Supplementary file 1 [file biosensors-14-00167-s001.zip › biosensors-2912484-Supplementary.pdf]

biosensors

Effect of Protection Polymer Coatings on the Performance of an Amperometric Galactose Biosensor in Human Plasma

Carina Figueiredo [1], Carolin Psotta [2,3], Kavita Jayakumar [4], Anna Lielpetere [5], Tanushree Mandal [4], Wolfgang Schuhmann [5], Dónal Leech [4], Magnus Falk [2], Marcos Pita [1], Sergey Shleev [2,3] and Antonio L. De Lacey [1,*]

1 Instituto de Catálisis y Petroleoquímica, CSIC, c/Marie Curie 2, 28049 Madrid, Spain; carina.felix@estudiante.uam.es (C.F.)

2 Department of Biomedical Science, Faculty of Health and Society, & Biofilms-Research Center for Biointerfaces, Malmo University, 205 06Malmö, Sweden; sergey.shleev@mau.se (S.S.)

3 Aptusens AB, 293 94 Kyrkhult, Sweden

4 School of Biological and Chemical Sciences & Ryan Institute, University of Galway, H91 TK33 Galway, Ireland; tanushreemandal2014@gmail.com (T.M.)

5 Analytical Chemistry-Center for Electrochemical Science (CES), Faculty of Chemistry and Biochemistry, Ruhr-University Bochum, 44791 Bochum, Germany; anna.lielpetere@ruhr-uni-bochum.de (A.L.); wolfgang.schuhmann@rub.de (W.S.)

* Correspondence: alopez@icp.csic.es; Tel.: +34-915854807

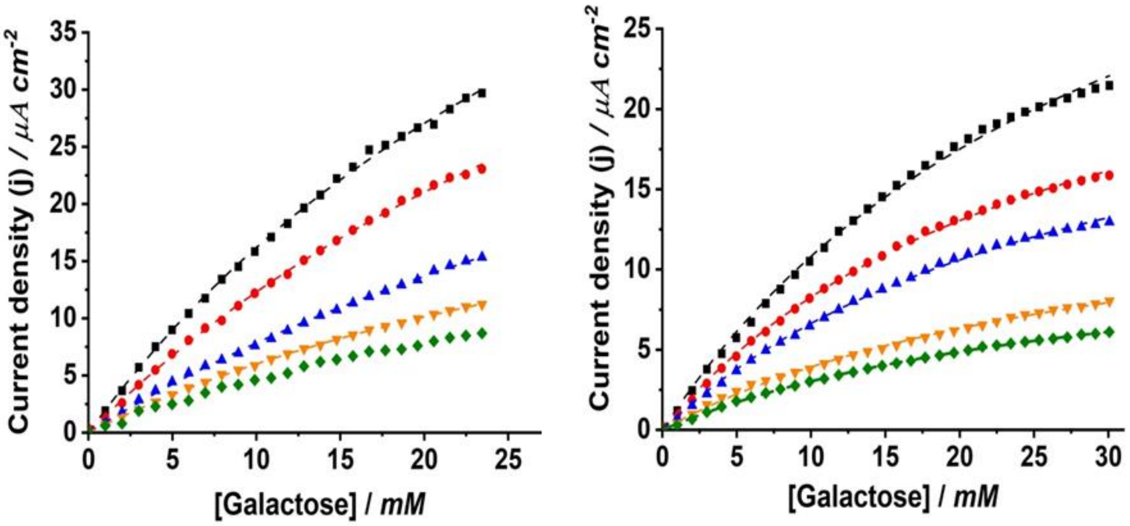

Figure S1. Current density dependence on galactose concentrations for uncoated (left graph) and coated GaOx-modified electrodes (right graph) measured by chronoamperometry measurements at +0.35 V vs. Ag/AgCl (3 M KCl) in 0.1 M phosphate buffer pH 7.0 after 1 (black), 2 (red), 3 (blue), 4 (orange) and 7 days (green) stored overnight in human plasma at 4ºC.
